# Nursing-sensitive quality indicators for quality improvement in Norwegian nursing homes – a modified Delphi study

**DOI:** 10.1186/s12913-023-10088-4

**Published:** 2023-10-06

**Authors:** Kjerstin Tevik, Anne-Sofie Helvik, Geir-Tore Stensvik, Marion S. Nordberg, Sigrid Nakrem

**Affiliations:** 1 Department of Public Health and Nursing, Faculty of Medicine and Health Sciences, Norwegian University of Science and Technology (NTNU), Trondheim, Norway; 2 The Norwegian National Centre for Ageing and Health, Vestfold Hospital Trust, Tønsberg, Norway; 3 Department of Geriatrics, St. Olavs Hospital, Trondheim University Hospital, Trondheim, Norway; 4 National Ageing Research Institute, Royal Melbourne Hospital, 34-54 Poplar Road, Victoria, 3050 Australia

**Keywords:** Clinical indicators, Consensus, Delphi process, Dementia, Geriatric nursing, interRAI LTCF, Long-term care, Quality indicators, Quality of healthcare, RAI-MDS

## Abstract

**Background:**

Use of nursing-sensitive quality indicators (QIs) is one way to monitor the quality of care in nursing homes (NHs). The aim of this study was to develop a consensus list of nursing-sensitive QIs for Norwegian NHs.

**Methods:**

A narrative literature review followed by a non-in-person, two-round, six-step modified Delphi survey was conducted. A five-member project group was established to draw up a list of nursing-sensitive QIs from a preliminary list of 24 QIs selected from Minimum Data Set (2.0) (MDS) and the international Resident Assessment Instrument for Long-Term Care Facilities (interRAI LTCF). We included scientific experts (researchers), clinical experts (healthcare professionals in NHs), and experts of experience (next-of-kin of NH residents). The experts rated nursing-sensitive QIs in two rounds on a seven-point Likert scale. Consensus was based on median value and level of dispersion. Analyses were conducted for four groups: 1) all experts, 2) scientific experts, 3) clinical experts, and 4) experts of experience.

**Results:**

The project group drew up a list of 20 nursing-sensitive QIs. Nineteen QIs were selected from MDS/interRAI LTCF and one (‘systematic medication review’) from the Norwegian quality assessment system IPLOS (‘Statistics linked to individual needs of care’). In the first and second Delphi round, 44 experts (13 researchers, 17 healthcare professionals, 14 next-of-kin) and 28 experts (8 researchers, 10 healthcare professionals, 10 next-of-kin) participated, respectively. The final consensus list consisted of 16 nursing-sensitive QIs, which were ranked in this order by the ‘all expert group’: 1) systematic medication review, 2) pressure ulcers, 3) behavioral symptoms, 4) pain, 5) dehydration, 6) oral/dental health problems, 7) urinary tract infection, 8) fecal impaction, 9) depression, 10) use of aids that inhibit freedom of movement, 11) participation in activities of interest, 12) participation in social activities, 13) decline in activities of daily living, 14) weight loss, 15) falls, and 16) hearing loss without the use of hearing aids.

**Conclusions:**

Multidisciplinary experts were able to reach consensus on 16 nursing-sensitive QIs. The results from this study can be used to implement QIs in Norwegian NHs, which can improve the quality of care.

**Supplementary Information:**

The online version contains supplementary material available at 10.1186/s12913-023-10088-4.

## Background

In Western countries, nursing home (NH) residents are a frail and complex population with high prevalence of dementia and physical diagnoses [1–6]. This frail NH-population demands a high quality of care [7]. One way to increase the quality of care in NHs is to develop quality indicators (QIs) [8]*.* QIs are used as a proxy measure that reflects the quality of care [7]*.* The purpose of QIs is also to highlight areas in an NH that may be performed poorly and where the quality could be improved [9]. Awareness of areas with low quality can lead to improved care processes and better outcomes among the NH residents [9–11]*.* Quality of care can be classified into three categories: 1) structure quality (i.e., facilities and number of qualified personnel), 2) process quality (what is done in the care process), and 3) outcome quality (effect of the care processes) [12, 13]*.*

International standards for NH care are not available [13]. In Norway, each municipality reports individual resident data from NHs annually in the IPLOS database (In Norwegian: Individbasert Pleie- og omsorgstatistikk: In English: Statistics linked to individual needs of care) [14]. IPLOS is well-suited for monitoring the service utilization on a national level, but is less suited for the management of clinical quality on an organizational level [15]. In other countries, several instruments have been developed to measure quality in NHs, such as the Resident Assessment Instrument Minimum Data Set (RAI-MDS, hereafter referred to as MDS) [16, 17], Assessing Care of Vulnerable Elders (ACOVE) [18, 19], Service measurement tool for healthcare (SERVQUAL) [20], Consumer Quality (CQ) index questionnaire [21], Impact of a quality improvement (IQUARE) questionnaire [22], and SeniorAlert [23]. Of these instruments, only the MDS has been rigorously tested in a number of reliability studies [17, 24–30] and assessed in validity studies [13, 29, 31–35].

MDS is a standardized data collection and monitoring system that was developed by the Center for Medicare and Medicaid in USA in 1987 and implemented in all NHs in USA in 1991 [13, 16, 17, 36]. The QIs were selected on the basis of clinical review, empirical analysis, and pilot testing of the feasibility of the QIs [17]. A revised version was developed in 1995 (MDS 2.0) [36] and included 35 QIs [7]. MDS seems to be a reliable [17, 24–30] and valid [26, 29, 31–35, 37–39] assessment tool, and NHs using MDS have improved the quality of care in several clinical areas [40, 41]. However, two systematic reviews [13, 36] have concluded that the reliability and validity of MDS is questioned in some areas, for example with underreporting of pain, falls, and depression, and should be interpreted with caution. A Delphi method could be used to select and prioritize the most important QIs from MDS [42]. Important QIs measure areas with high volume (aspects of care that occur frequently) and high risk (aspects of care that involve risk), and should be sensitive to detect differences in care [13, 43].

Use of MDS facilitates standardized routine assessment and documentation of NH residents. Further, it enables comparison across NHs and is important for clinical planning and decision making [7, 9, 10]. Assessment of NH residents with MDS is completed at the time of admission and every third month thereafter or when there is significant change in the resident’s health status [17]. When comparing facilities, the QIs in MDS are risk-adjusted, which means that the differences in the risk profiles of resident populations are taken into account [7], as the QIs are intended to detect differences in quality of care and not differences in patient characteristics [13]. Today, MDS is mandated by central government and fully integrated on a national level in several countries (i.e., USA, Canada, and Iceland) [7, 8, 16].

In 1992 the international collaboration network interRAI was established to apply the MDS in NHs in other countries. The interRAI collaboration network consists of clinicians and researchers from more than 35 countries. They developed the international Resident Assessment Instrument for Long-Term Care Facilities (interRAI LTCF, hereafter referred to as interRAI), which included MDS 2.0 [44–46]. The latest version of interRAI includes more than 30 QIs [47], and in Norway we have a Norwegian version [46]. However, the use of interRAI has not been implemented in Norway on a national level, and has only been used for research. The Norwegian *Knowledge Centre for* *the* *Health Services* has recommended that QIs from MDS should be developed and included in a national quality system for the primary health services in Norway [48]. It could be useful to conduct a Delphi study [49] before implementing QIs from MDS in Norway. The aim of a Delphi study would be to reach consensus regarding QIs that could be important to implement in the Norwegian NH-setting [42]. Consensus regarding QIs may be considered as a measure of face validity. Face validity is a subjective judgment of a construct and is often considered as the weakest form of validity [50], but will be an important first step in the implementing process of QIs. To implement QIs, it is important to engage and involve different stakeholders who are interested in quality improvement, such as professionals (i.e., researchers and healthcare professionals) and consumers (i.e., NH residents and their family members) [7, 9]. The Delphi method has been used in several studies addressing QIs in NHs [6, 7, 51–57]. However, few studies have used a Delphi method to select and prioritize QIs from MDS [7]. Further, MDS was developed by researchers in USA [16, 17], and it may be important to conduct a Norwegian Delphi study, as the selection and prioritization of QIs may differ between countries.

As already mentioned, MDS consists of over 30 QIs and not all of them are sensitive to clinical practice [7]. To handle a more manageable list of QIs, we have chosen to focus on nursing-sensitive QIs [7]. In NHs, nursing care is the common service provided for the residents [13], and nursing-sensitive QIs can be defined as “measure of changes in health status upon which nursing care may have direct influence” (ICN, 2001) [13, 58]. By focusing on nursing-sensitive QIs, we assume that these indicators might have the greatest potential for functional improvement and slowing the functional decline among residents in NHs [7]. Thus, the aim of this study was to use a modified Delphi method [59] to describe the process used for selecting, rating, and developing a final consensus list of nursing-sensitive QIs from the Norwegian version of interRAI [46]. This list of QIs could be implemented in Norwegian NHs to improve the quality of care.

## Methods

### Design

A narrative literature review followed by a non-in-person, two-round, six-step modified Delphi survey was used to collect data.

### The Norwegian nursing home context

In Norway, there are approximately 950 NHs, comprising 39,200 beds [60, 61]. Ninety-one percent of the NHs are owned and run by the municipalities [61]. The Norwegian NHs are designed for residents who require a high level of medical care and assistance with daily activities [62]. Sixty-six percent of the residents are above 80 years of age, and about eighty-four percent have dementia [1]. Physical diagnoses such as cardiovascular, musculoskeletal, and endocrine, nutritional, and metabolic diseases are common in Norwegian residents with and without dementia [2]. A physician is responsible for the medical treatment, and a Registered Nurse (RN) is responsible for the nursing [63]. The NHs provide round-the-clock care [63] by RNs (31% of the staff), Licensed Practical Nurses (LPNs) (45% of the staff), and nursing assistants with no formal healthcare education (24% of the staff) [44].

### The MDS instrument and the Norwegian version of interRAI

As already mentioned, in the present study we used the Norwegian version of interRAI, which includes the revised version of MDS (MDS 2.0) [44, 46]. MDS assesses the process and outcome quality of care and not the structure quality [17]. The QIs in MDS are organized into 12 domains focusing on both physical and psychosocial individual factors. The domains are accidents, behavioral and emotional patterns, cognitive function, elimination and continence, infection control, nutrition and eating, physical function, psychotropic drug use, quality of life, sensory function and communication, skin care, and clinical management (i.e., medical treatment) [8, 17]. The Norwegian version of interRAI has been used in several studies in Norwegian NHs [25, 44, 64–66].

### Modified Delphi method

The Delphi method is a multistage process [49] characterized by anonymity, iteration, and controlled feedback of the results to a group of ‘experts’ [59, 67]. The experts are the participants included in the Delphi study. In our study, we included professional experts (researchers and healthcare professionals in NHs) and experts of experience (next-of-kin of NH residents). The aim was to obtain group consensus on the experts’ opinions regarding nursing-sensitive QIs with the use of series of structured questionnaires, which are referred to as rounds [49]. Two or three rounds are preferred in a Delphi study [49]. In the present study, we selected a priori to use a two-round Delphi method. After each round, the participants received feedback of the results. The experts did not meet each other face-to-face during the Delphi process [49].

Traditionally, the first round in the Delphi method begins with an open-ended questionnaire to generate ideas around the topic of interest [56, 59]. However, we chose to use a modified Delphi method, where we started the process with a narrative literature review. This is regarded as an acceptable and common modification of the Delphi process [59].

**Fig. 1:**
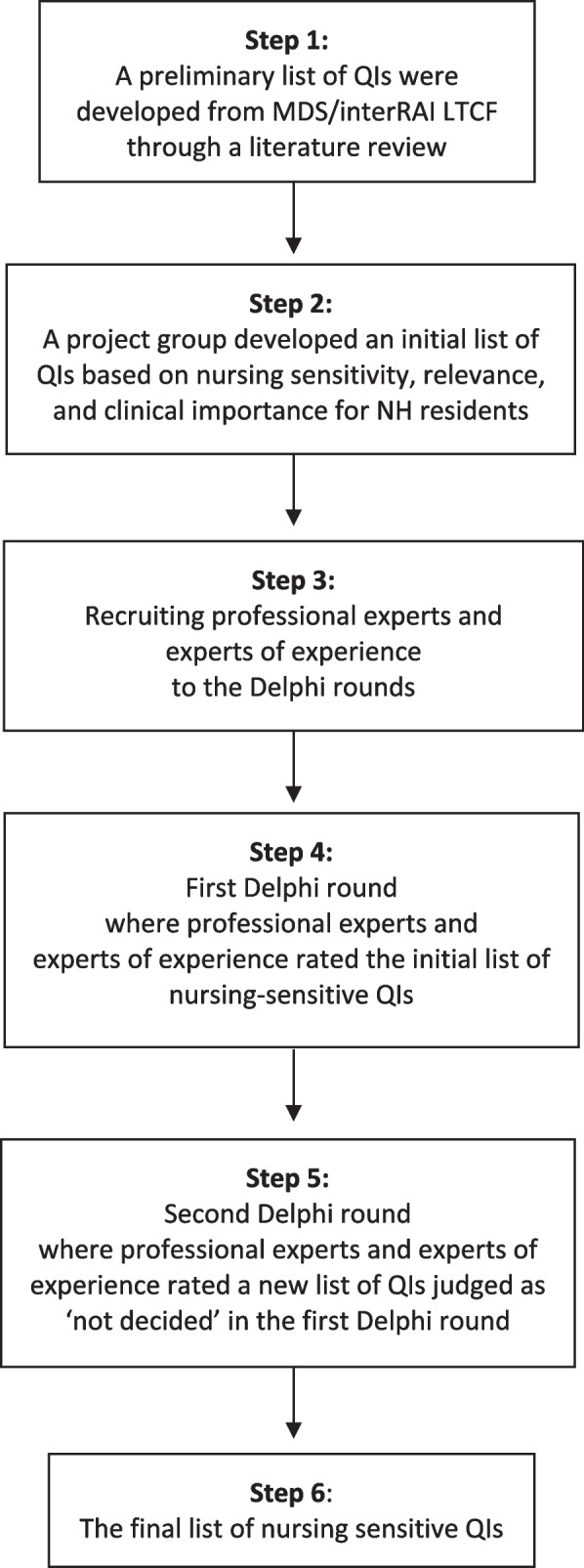
Flow diagram showing the six-step modified Delphi process. Abbreviations: interRAI LTCF = international Resident Assessment Instrument for Long-Term Care Facilities; MDS = Minimum Data Set; NH = Nursing Home; QI = Quality Indicator

This study used a six-step process to identify a final list of nursing-sensitive QIs. Figure 1 shows the stepwise procedure in a flow diagram. Each step will be described in more detail in the following section.

### Step 1: narrative literature review and the preliminary list of quality indicators

A narrative literature review was conducted to identify studies that had examined the use of MDS or interRAI in NHs. The databases Medline, PsycInfo, Web of Science, and Embase were searched for articles published between 1985 and 2020 by author SN. The keywords that were used are found in Additional file 1. A total of 5,530 records were identified and uploaded to the Rayyan web application that helped us expedite the initial screening of titles and abstracts [68]. Additional file 2 presents the PRISMA flow diagram [69], which gives an overview of the search strategy and the inclusion process of the studies. The full texts of 303 studies were considered, and 72 studies were found relevant for this study. A preliminary list of QIs from MDS or interRAI was developed by the authors KT and SN based on this review.

### Step 2: the project group and the initial list of nursing-sensitive quality indicators

A five-member project group was established. All were RNs with both clinical and academic research experience from NHs or geriatric units. Three RNs had a doctoral degree, and two were Advanced Clinical Nurses in geriatric nursing with a master’s degree. The project group evaluated the preliminary list of QIs detected in the review, and drew up an initial list of QIs using several inclusion and exclusion criteria. The QIs should be nursing-sensitive, which means that changes in the residents’ health status are directly influenced by nursing care (ICN, 2001) [13, 58], and the QIs should have high prevalence, measure areas of high risk, and be sensitive to detect differences in care [13, 43]. The initial list of nursing-sensitive QIs consisted of process and outcome indicators [17, 46]. The project group members did not participate in the actual Delphi rounds but reviewed the results from the first and second Delphi round and worked out a new list of nursing-sensitive QIs for which consensus was reached after each round [56, 57, 70]. A total of three project group meetings were held.

### Step 3: recruiting experts to the Delphi rounds

We recruited both professional experts (scientific experts and clinical experts) and experts of experience [71]. The criterion for being a scientific expert was to be (or to have been) a scientist in the field of elderly care, NH, and quality. The criterion for being a clinical expert was to be an RN, Intellectual Disability Nurse, or an LPN working at an NH with at least one year of clinical experience. Lastly, the criterion for being an expert of experience was to be the next-of-kin of an NH resident.

We obtained names of potential experts from contact persons for the ‘Aging and Older Persons Health’ research group at the Department of Public Health and Nursing (ISM), Norwegian University of Science and Technology (NTNU), and from NH managers in Mid-Norway.

We invited a total of 25 researchers, 25 healthcare professionals, and 16 next-of-kin to participate in the first Delphi round. Scientific experts were recruited from eleven universities and three research institutions from the whole of Norway. Healthcare professionals and next-of-kin were recruited from four different NHs in Mid-Norway.

### Step 4: first Delphi round

Data collection from the professional experts in the Delphi rounds was handled by electronic questionnaires created with nettskjema.no, which is a survey solution developed and hosted by the University of Oslo, Norway [72]. In advance of the Delphi rounds, electronic questionnaires were pilot-tested [42] among three RNs with scientific and/or clinical experience of NHs. The pilot testing revealed that the questionnaires were suitable. The electronic questionnaire was distributed to the professional experts via email. Experts of experience (next-of-kin) could choose whether they wanted to respond to an electronic questionnaire via email or a postal paper questionnaire.

The first questionnaire included the initial list of nursing-sensitive QIs, and the experts were asked to rate the QIs according to the importance to nursing practice on a Likert scale from 1 (not important at all) to 7 (very important) [59, 71]. A higher score indicated a higher level of importance (see Table 1).

**Table 1:** Criterion for rating quality indicators using a 7-point Likert scale

Criterion	Definition	Score 0–2	Score 3–5	Score 6–7
Importance	If implemented, the care process will affect the overall quality of NH-care	Not important for evaluating nursing care quality	Uncertain importance for nursing care quality	Clearly important for evaluating or providing quality of nursing care

*NH* Nursing Home

References: Røsvik et al. 2020 [71], Saliba et al. 2002 [55]

### Step 5: second Delphi round

In the second Delphi round, the participants who had responded to the first questionnaire received a new electronic [72] or postal paper questionnaire with a list of nursing-sensitive QIs for which consensus was not reached in the first Delphi round.

We gave the participants two weeks to respond to the questionnaire in each round. One reminder email or letter was sent to non-responders after each round.

### Step 6: the final list of nursing-sensitive quality indicators

After the second round, the project group worked out the final consensus list of nursing-sensitive QIs. The final list will be sent to the experts when the results from this study are published.

## Analyses

The data was analyzed using Microsoft Excel 2018 [73] and IBM SPSS version 27 [74].

Consensus and level of agreement of the QIs were based on the median value and level of dispersion [59, 71]. Analyses were conducted for four groups: 1) all experts, 2) scientific experts, 3) clinical experts, and 3) experts of experience.

In the first Delphi round, a consensus was achieved for a QI as important if the median score was 6 or 7; it was undecided with a median score of 3, 4, or 5; and regarded as not important if the median score was 1 or 2 [71]. Regarding dispersion, consensus was reached if the quartile deviation (interquartile range divided by 2) was ≤ 5 and ≥ 75% of the ratings of a QI were within two adjoining values (score 6 and 7) [71]. If a consensus was achieved for a QI in every expert group in the first Delphi round, it was accepted without resubmission for a second round. QIs judged as not important in the first round (median score 1 or 2) were rejected and not resubmitted for a second round. QIs judged as undecided in one or more expert groups in the first round (median score 3, 4, or 5; quartile deviation > 5; or < 75% of the ratings were within two adjoining values – score 6 and 7), were resubmitted for a second round [71].

Participants in the second Delphi round received a summary of the median score and the lowest and highest score for each QI for the whole group and their personal score [49, 56]. Consensus was achieved for a QI after the second round if consensus was reached in the ‘all expert’ group [71]. Consensus was based on the same threshold values as described for the first Delphi round. All other QIs were rejected.

The final list of nursing-sensitive QIs was ranked according to the results from the first and second rounds. The QIs were ranked according to whether or not consensus had been achieved, the highest median value, the highest percent of the rating between two adjoining values (6 or 7), the lowest interquartile range, and the range values.

## Results

### The preliminary list and the initial list of nursing-sensitive quality indicators

Based on the literature review, a preliminary list of 24 QIs was selected from MDS/interRAI by the authors KT and SN (see Additional file 3 and Table 2). The project group rejected six of the 24 QIs in the preliminary list and added two new QIs (see Table 2). These QIs were ‘hearing loss without the use of hearing aids’ and ‘systematic medication review’. InterRAI includes a subjective measure of ‘hearing loss without the use of hearing aids’ [46]. ‘Systematic medication review’ is not included in interRAI but is part of the Norwegian quality assessment system IPLOS [14]. In total, the initial list consisted of 20 nursing-sensitive QIs, which are described and defined in Table 2.

**Table 2:** The preliminary and the initial list of nursing-sensitive quality indicators

QI number	Preliminary list of QIs identified from a literature review	Initial list of nursing-sensitive QIs after the project group decision^a^	Description/definition of the QIs in the initial list^b^
1	Incidence of cognitive impairment	Rejected	
2	Prevalence of symptoms of depression	Accepted/modified Proportion of residents with symptoms of depression in past three days with and without treatment	Examples of symptoms of depression: 1) The resident has little interest or pleasure in things he/she usually enjoys; 2) The resident is anxious or restless; 3) The resident is sad, depressed, or thinks everything is hopeless
3	Prevalence of symptoms of depression without antidepressant therapy	Included in the forementioned QI	
4	Prevalence of behavioral symptoms affecting others	Accepted/modified Proportion of residents with behavioral symptoms in past three days affecting others	Examples of behavioral symptoms: 1) Walking around without seeing their own needs or security: 2) Verbally aggressive; 3) Physically aggressive; 4) Socially deviant behavior; 5) Sexually deviant behavior or undressing in public; 6) Opposes care
5	Prevalence of little or no social activity	Accepted/modified Proportion of residents with a certain degree of participation in social activities	Examples of social activities: 1) An activity the resident has been interested in for a long time; 2) Visit from an old friend or family; 3) Telephone or email contact with an old friend or family
6	Prevalence of little or no activity	Accepted/modified Proportion of residents with a certain degree of participation in activities of interest for them	Examples of activities: Cards/toys/puzzles; PC activities; talking on the phone; needlework; dancing, discussions, or conversations; exercising; gardening activities; helping others; music; pets; reading; writing; crossword puzzles; spiritual or religious activity; traveling or shopping; walking or driving a wheelchair outdoors; watching TV or listening to the radio
7	Incidence of decline in ADL	Accepted/modified Proportion of residents with worse ADL status compared to 90 days ago	ADL means ‘Activities of Daily Living’ Examples of ADL activities: Bathing; personal hygiene; dressing and undressing; walking; moving; using the toilet; mobility in bed; and how the resident eats and drinks
8	Prevalence of physical restraints	Accepted/modified Proportion of residents with the use of aids that inhibit freedom of movement	Use of aids that inhibit freedom of movement means involuntary use of coercive devices that inhibit normal movement. Involuntary use means without consent from the resident Examples of aids that inhibit freedom of movement: 1) Bed rails that are completely up on all open sides of the bed; 2) Fixation of the resident in the chair with harness or belt; 3) Obstacles leading to the resident being unable to get up from the chair
9	Prevalence of falls	Accepted/modified Proportion of residents with falls in past 30 days	No further description/definition
10	Prevalence of bedfast residents	Accepted/modified Proportion of bedfast residents	A bedfast resident is defined as one who lies in bed or sits in an armchair/recliner for 22 of the 24 h of the day
11	Prevalence of pressure ulcers	Accepted/modified Proportion of residents with pressure ulcers	Pressure ulcers are caused by prolonged pressure on a skin area over a bone prominence Pressure ulcers can be graded in severity: 1) Area of skin redness; 2) Partial loss of skin layer; 3) Deep skin craters; 4) Full thickness skin loss exposing muscle and/or bone; 5) Necrotic tissue dominates
12	Prevalence of bladder/bowel incontinence	Accepted/modified Proportion of residents with bladder and bowel incontinence	Bladder and bowel incontinence means loss of urine and bowel function
13	Prevalence of fecal impaction	Accepted/modified Proportion of residents with fecal impaction	Fecal impaction means no stools within three days or problems with hard stools
14	Prevalence of toilet assistance program	Accepted/modified Proportion of residents with established toilet routines	Established toilet routines can be planned toilet visits where employees accompany residents to the toilet at set times
15	Prevalence of indwelling catheters	Accepted/modified Proportion of residents with an indwelling urinary catheter	A urinary catheter is a hollow and thin flexible tube inserted through the urethra into the bladder. An indwelling catheter refers to a catheter that is left in the bladder all the time
16	Prevalence of urinary tract infection	Accepted/modified Proportion of residents with urinary tract infection in past 30 days	Urinary tract infection means bacterial infection in the lower or upper urinary tract (bladder and renal pelvis)
17	Prevalence of pain	Accepted/modified Proportion of residents with pain without adequate pain treatment	No further description/definition
18	Prevalence of weight loss	Accepted/modified Proportion of residents with weight loss	Weight loss is defined as a weight loss of 5% or more in the past 30 days, or a weight loss of 10% or more in the past 180 days Example 1: A weight loss of 4 kg in the past 30 days from 65 to 61 kg means a weight loss of approximately 6% Example 2: A weight loss of 7 kg in the past 180 days from 65 to 58 kg means a weight loss of approximately 11%
19	Prevalence of tube feeding	Rejected	
20	Prevalence of dehydration	Accepted/modified Proportion of residents with dehydration	Dehydration can be defined as: 1) Insufficient fluid intake, less than 1000 ml per day; 2) Fluid loss greater than fluid intake
21	Prevalence of oral and dental health problem	Accepted/modified Proportion of residents with dental and/or oral problems	Dental or oral problems are assessed based on whether the resident has: 1) Dentures and/or removable bridge; 2) Not intact natural teeth; 3) Pain/discomfort in mouth or face; 4) Dry mouth; 5) Ingestion problems; 6) Inflamed or bleeding gums
22	Prevalence of use of 9 or more different medications	Rejected	
23	Prevalence of antipsychotic use in the absence of psychotic and related conditions	Rejected	
24	Prevalence of antianxiety/hypnotic use	Rejected	
**New**		**Suggestion from the project group** Proportion of residents with hearing loss without the use of hearing aids^c^	No further description/definition
**New**		**Suggestion from the project group** Proportion of residents who have had a systematic medication review in past year or when needed^d^	A medication review is a systematic procedure to ensure the quality of the resident’s drug use. The medication review can be carried out in an interdisciplinary team consisting of a physician, a registered nurse, and/or a pharmacist^d^

*ADL* Activities of Daily Living, *PC* Personal Computer, *QI* Quality Indicator

^a^The QIs are ranked in the order in which they are described in the questionnaire to the participants in the Delphi rounds

^b^The description/definition of each QI was included in the questionnaire distributed to the participants in the Delphi rounds

^c^A quality indicator recommended by the project group. ‘Hearing loss without the use of hearing aids’ can be assessed subjectively in MDS/interRAI [46]

^d^A quality indicator not included in MDS/interRAI but recommended as a quality indicator by the project group. ‘Systematic medication review’ is included as a quality indicator in the Norwegian quality assessment system (IPLOS) [14]

References: Zimmerman et al. (2003): Improving nursing home quality of care through outcomes data: The MDS quality indicators [11]; Morris et al. (2012): interRAI Long-Term Care Facilities (LTCF). Assessment Form and User’s Manual. Version 9.1. Norwegian Version. Washington, DC: interRAI [46]

### The participants in the Delphi rounds

In total, 13 researchers, 17 healthcare professionals, and 14 next-of-kin (*N* = 44) responded to the questionnaire in Delphi round 1, and eight researchers, 10 healthcare professionals, and 10 next-of-kin (*N* = 28) responded to the questionnaire in Delphi round 2 (Table 3). The response rate was 66.7% and 63.6% in the first and second round, respectively (Table 3). Three next-of-kin responded to a postal paper questionnaire in the first round, and one next-of-kin in the second round. The participants were recruited and included in the study from November 2021 through August 2022. The characteristics of the participants in every expert group are shown in Table 4.

**Table 3:** Number of participants in the first and second Delphi rounds

		**Round 1** **Professional experts:** Scientific experts: Researchers^a^	**Round 1** **Professional experts:** Clinical experts: Healthcare professionals^b^	**Round 1** **Experts of experience:** Next-of-kin of NH residents	**Total Round 1**
**Invited**	n	25	25	16	66
**Responded**	n	13	17	14	44
**Response rate**	%	52.0	68.0	87.5	66.7
		**Round 2** **Professional experts:** Scientific experts: Researchers^a^	**Round 2** **Professional experts:** Clinical experts: Healthcare professionals^b^	**Round 2** **Experts of experience:** Next-of-kin of NH residents	**Total Round 2**
**Invited**	n	13	17	14	44
**Responded**	n	8	10	10	28
**Response rate**	%	61.5	58.8	71.4	63.6

*Abbreviations*: *n* number, *NH* Nursing Home

^a^Researchers in elderly care and quality in NHs

^b^Registered Nurses and Licensed Practical Nurses working in NHs

**Table 4:** Characteristics of the participants

	**Professional experts**	
	Scientific experts: Researchers	
	Round 1 (*n* = 13) n (%)	Round 2 (*n* = 8) n (%)
Gender:		
Female	13 (100)	8 (100)
Age group		
40–49 years	4 (30.8)	4 (50.0)
50–59 years	7 (53.8)	3 (37.5)
60–69 years	1 (7.7)	0
70–79 years	1 (7.7)	1 (12.5)
Profession:		
PhD Fellow	1 (7.7)	1 (12.5)
Associate Professor	5 (38.5)	3 (37.5)
Professor	4 (30.8)	2 (25.0)
Scientist	1 (7.7)	1 (12.5)
Other title	2 (15.3)	1 (12.5)
Education		
MSc	1 (7.7)	1 (12.5)
PhD	11 (84.6)	7 (87.5)
Other academic degree	1 (7.7)	0
	Clinical experts: Healthcare professionals	
	Round 1 (*n* = 17) n (%)	Round 2 (*n* = 10) n (%)
Gender		
Female	16 (94.1)	9 (90.0)
Age group		
18–29 years	2 (11.8)	0
30–39 years	4 (23.5)	2 (20.0)
40–49 years	5 (29.4)	3 (30.0)
50–59 years	4 (23.5)	3 (30.0)
60–69 years	2 (11.8)	2 (20.0)
Profession		
LPN	2 (11.8)	0
RN	8 (47.1)	5 (50.0)
Advanced Clinical Nurse	1 (5.9)	0
Clinical Unit Manager	3 (17.6)	2 (20.0)
NH Manager	3 (17.6)	3 (30.0)
Education		
Vocational	2 (11.8)	0
Bachelor	14 (82.3)	10 (100)
MSc	1 (5.9)	0
Working unit in an NH		
Regular care unit	10 (58.8)	5 (50.0)
Special care unit	5 (29.4)	4 (40.0)
Several units	2 (11.8)	1 (10.0)
Number of years working in an NH		
1–4 years	4 (23.5)	2 (20.0)
5–9 years	1 (5.9)	1 (10.0)
10–14 years	3 (17.7)	3 (30.0)
15–19 years	4 (23.5)	1 (10.0)
≥ 20 years	5 (29.4)	3 (30.0)
	**Experts of experience**	
	Next-of-kin of NH residents	
	Round 1 (*n* = 14) n (%)	Round 2 (*n* = 10) n (%)
Gender:		
Female	10 (71.4)	8 (80.0)
Age group		
40–49 years	1 (7.2)	0
50–59 years	5 (35.7)	4 (40.0)
60–69 years	3 (21.4)	3 (30.0)
70–79 years	3 (21.4)	1 (10.0)
80–89 years	2 (14.3)	2 (20.0)
Relationship to the resident^a^		
Spouse/cohabitant/partner	4 (30.8)	3 (30.0)
Son/daughter	7 (53.8)	5 (50.0)
Other relationship	2 (15.4)	2 (20.0)
Numbers of years residents have lived in an NH^a^		
< 1 year	2 (15.4)	2 (20.0)
1–2 years	3 (23.1)	1 (10.0)
3–4 years	7 (53.8)	6 (60.0)
5–9 years	1 (7.7)	1 (10.0)

*LPN* Licensed Practical Nurse, *MSc* Master of Science, *n* number, *NH* Nursing Home, *PhD* Philosophiae Doctor, *RN* Registered Nurse

^a^1 missing in the first Delphi round

In the first round, the participating scientists came from six different universities and two research institutions from the whole of Norway. Participating healthcare professionals and next-of-kin came from four different NH settings in Mid-Norway. The NHs were located in small (< 6,000 inhabitants) (three NHs) and medium-sized (6,000–20,000 inhabitants) (one NH) municipalities [75, 76]. All NHs were non-profit and run and owned by the municipalities.

### First Delphi round

Table 5 presents the rating of the 20 QIs for the whole group and for every expert group in the first Delphi round. In total, consensus was achieved for nine QIs as ‘important’, and 11 QIs were ‘undecided’ in either one or more expert group. None of the QIs were stated as ‘not important’. Of the 11 QIs for which consensus was not achieved, seven QIs had too low a median value and four had both too low a median value and too high levels of dispersion (Table 5).

**Table 5:** Results for 20 nursing-sensitive quality indicators in Delphi round 1

First round				
Median / % of the ratings between two adjoining values (6–7) / Quartile deviation / Range (minimum and maximum score)				
**Quality indicator**^**a**^	**All experts**	**Scientific experts:** Researchers	**Clinical experts:** Healthcare professionals	**Experts of experience: Next-of-kin**
1. Symptoms of depression	**7/ 88.4/ 0.5**/ 3-7^b^	**6/ 77.0/ 1.0**/ 3–7	**7/ 94.1/ 0.5**/ 4–7	**7.0/ 92.3/ 0.5**/ 4-7^b^
2. Behavioral symptoms affecting others	**7/ 93.1/ 0.5**/ 4–7	**7/ 92.3/ 0.5**/ 5–7	**7/ 100/ 0.5**/ 6–7	**7.0/ 85.7/ 0.5**/ 4–7
3. Participation in social activities	**6/ 75.0/ 1.0**/ 2–7	*6/ 69.3/ 1.0/* 4–7	**6/ 82.4/ 0.5**/ 5–7	*6.0/ 71.5/ 1.0*/ 2–7
4. Participation in activities of interest	**6/ 81.8/ 0.5**/ 4–7	*6/ 69.3/ 1.0/* 4–7	**6/ 88.3/ 0.5**/ 5–7	**7.0/ 85.7/ 0.5**/ 4–7
5. Decline in ADL	*6/ 68.2/ 1.0*/ 2–7	*6/ 53.9/ 1.5/* 3–7	**6/ 76.5/ 1.0**/ 2–7	*6.5/ 71.4/ 1.0/* 3–7
6. Use of aids that inhibit freedom of movement	**7/ 77.5/ 0.5**/ 2-7^c^	**7/ 92.3/ 0.5**/ 4–7	**7/ 82.3/ 0.5**/ 3–7	*5.5/ 50.0/ 1.0/* 2-7^c^
7. Falls	**6/ 81.4/ 0.5**/ 4-7^b^	**6/ 77.0/ 1.0/** 4–7	**7/ 82.3/ 0.5**/ 5–7	**6.0/ 84.7/ 0.5/** 4-7^b^
8. Bedfast residents	*5/ 41.9/ 1.0/* 1-7^b^	*4/ 38.5/ 1.0/* 3–7	*5/ 41.4/ 0.5*/ 1–7	*5.0/ 46.2/ 1.0/* 1-7^b^
9. Pressure ulcers	**7/ 95.2/ 0.0**/ 5-7^d^	**7/ 92.3/ 0.5**/ 5–7	**7/ 100/ 0.0**/ 6–7	**7.0/ 91.7/ 0.5**/ 5-7^d^
10. Bladder and bowel incontinence	*5/ 43.2/ 1.5/* 1–7	*6/ 53.9/ 1.5/* 3–7	*5/ 23.5/ 0.5*/ 1–7	*6.0/ 57.2/ 1.0/* 3–7
11. Fecal impaction	**7/ 79.1/ 0.5**/ 3-7^b^	*6/ 69.3/ 1.5/* 3–7	**7/ 94.1/ 0.5**/ 5–7	*6.0/ 69.3/ 1.0/* 5-7^b^
12. Established toilet routines	*6/ 68.1/ 1.0*/ 1–7	*6/ 53.9/ 1.5/* 1–7	**6/ 82.4/ 0.5**/ 5–7	*6.5/ 64.3/ 1.0/* 3–7
13. Indwelling urinary catheter	*5/ 48.8/ 1.0*/ 3-7^c^	*5/ 46.2/ 1.0/* 4–7	*5/ 41.4/ 1.0*/ 4–7	*6.0/ 63.7/ 1.5/* 3-7^c^
14. Urinary tract infection	**6/ 81.4/ 0.5**/ 3-7^b^	**7/ 100/ 0.5/** 6–7	*6/ 64.7/ 1.0*/ 3–7	**6.0/ 84.7/ 0.5**/ 5-7^b^
15. Pain	**7/ 93.0/ 0.0**/ 4-7^b^	**7/ 100/ 0.5**/ 6–7	**7/ 100/ 0.0**/ 6–7	**7.0/ 76.9/ 0.5**/ 4-7^b^
16. Weight loss	**6/ 86.4/ 0.5**/ 5–7	**6/ 92.3/ 0.5/** 5–7	**6/ 94.1/ 0.5**/ 5–7	*6.0/ 71.5/ 1.0/* 5–7
17. Dehydration	**7/ 90.9/ 0.5**/ 5–7	**7/ 100/ 0.5**/ 6–7	**7/ 88.2/ 0.5**/ 5–7	**7.0/ 85.7/ 0.5/** 5–7
18. Oral and/or dental health problem	**7/ 90.7/ 0.5**/ 4-7^b^	**7/ 84.6/ 0.5**/ 4–7	**7/ 100/ 0.5**/ 6–7	**7.0/ 84.6/ 0.5/** 5-7^b^
19. Hearing loss without the use of hearing aids	**6/ 79.1/ 0.5**/ 4-7^b^	**6/ 77.0/ 1.0/** 4–7	**6/ 76.5/ 1.0**/ 4–7	**7.0/ 84.6/ 0.5/** 4-7^b^
20. Systematic medication review	**7/ 97.8/ 0.0**/ 4–7	**7/ 92.3/ 0.0**/ 4–7	**7/ 100/ 0.0**/ 6–7	**7.0/ 100/ 0.5**/ 6–7

**Bold font**: consensus

*Italics:* NO consensus/resubmitted to the second Delphi round

*Abbreviation*: *ADL* Activities of Daily Living

^a^The QIs are ranked in the order in which they are described in the questionnaire to the participants in the first Delphi round

^b^Missing: *n* = 1

^c^Missing: *n* = 3

^d^Missing: *n* = 2

### Second Delphi round

Table 6 shows the rating of the 11 QIs for the whole group and for every expert group in the second Delphi round. Of the 11 QIs regarded as ‘undecided’ in the first Delphi round, seven QIs reached consensus as ‘important’ in the ‘all expert group’ in the second round. Of the four QIs for which consensus was not achieved, one had too low a median value and three had both too low a median value and too high levels of dispersion (Table 6).

**Table 6:** Results for 11 nursing-sensitive quality indicators in Delphi round 2

Second round				
Median / % of the ratings between two adjoining values (6–7) / Quartile deviation / Range (minimum and maximum score)				
**Quality indicator**^**a**^	**All experts**	**Scientific experts:** Researchers	**Clinical experts:** Healthcare professionals	**Experts of experience:** Next-of-kin
1. Participation in social activities	**6.5/ 89.3/ 0.5/** 4–7	**6.0/ 87.5/ 0.5/** 4–7	**6.0/ 100/ 0.5/** 6–7	**6.5/ 80.0/ 0.5/** 5–7
2. Participation in activities of interest	**6.5/ 96.4/ 0.5/** 4–7	**6.0/ 87.5/ 0.5/** 4–7	**6.5/ 100/ 0.5/** 6–7	**7.0/ 100/ 0.5/** 6–7
3. Decline in ADL	**6.5/ 82.1/ 0.5/** 4–7	**6.5/ 75.0/ 1.0/** 4–7	**6.5/ 90.0/ 0.5/** 5–7	**6.5/ 80.0/ 0.5/** 4–7
4. Use of aids that inhibit freedom of movement	**7.0/ 85.2/ 0.5/** 4-7^b^	**7.0/ 100/ 0.5/** 6–7	**7.0/ 80.0/ 0.5/** 5–7	**6.0/ 77.7/ 1.0/** 4-7^b^
5. Bedfast residents	*5.0/ 48.1/ 0.5/* 1-7^b^	*5.0/ 37.5/ 1.0/* 4–6	*6.0/ 60.0/ 1.5/* 1–7	*5.0/ 44.4/ 1.0/* 4-7^b^
6. Bladder and bowel incontinence	*5.5/ 50.0/ 1.0/* 1–7	*5.5/ 50.0/ 1.5/* 3–7	*5.0/ 30.0/ 1.0/* 1–7	*6.0/ 70.0/ 1.5/* 4–7
7. Fecal impaction	**7.0/ 89.3/ 0.5/** 3–7	*6.5/ 62.5/ 1.0/* 3–7	**7.0/ 100/ 0.5/** 6–7	**7.0/ 100/ 0.5/** 6–7
8. Established toilet routines	*6.0/ 74.0/ 1.0/* 3-7^b^	*6.0/ 62.5/ 1.5/* 3–7	**6.0/ 80.0/ 0.5/** 5–7	**6.0/ 77.7/ 1.0/** 5-7^b^
9. Indwelling urinary catheter	*5.0/ 39.3/ 1.0/* 3–7	*4.5/ 37.5/ 1.0/* 3–7	*5.0/ 30.0/ 1.0/* 4–7	*5.5/ 50.0/ 1.0/* 3–7
10. Urinary tract infection	**7.0/ 89.3/ 0.5/** 4–7	**7.0/ 100/ 0.5/** 6–7	*6.0/ 70.0/ 1.0/* 4–7	**7.0/ 100/ 0.5/** 6–7
11. Weight loss	**6.0/ 92.9/ 0.5/** 4–7	**6.5/ 87.5/ 0.5/** 4–7	**6.5/ 90.0/ 0.5/** 5–7	**6.0/ 100/ 0.5/** 6–7

**Bold font**: consensus

*Italics:* NO consensus

*Abbreviation*: *ADL* Activities of Daily Living

^a^The QIs are ranked in the order in which they are described in the questionnaire to the participants in the second Delphi round

^b^Missing: *n* = 1

### Ranking order and final list of nursing-sensitive quality indicators

Of the initial list of 20 QIs, consensus was reached for 16 nursing-sensitive QIs as important in the two Delphi rounds in the ‘all expert group’ (Table 7). Systematic medication review was ranked as the most important nursing-sensitive QI, followed by pressure ulcers, behavioral symptoms affecting others, pain, and dehydration (Table 7). The rejected QIs for which consensus was not achieved were established toilet routines, bladder and bowel incontinence, bedfast residents, and indwelling urinary catheter.

**Table 7:** Final consensus list of nursing-sensitive quality indicators among all experts

All experts	
**Ranking**^a^** order of the quality indicators**	
1	**Systematic medication review**
2	**Pressure ulcers**
3	**Behavioral symptoms affecting others**
4	**Pain**
5	**Dehydration**
6	**Oral and/or dental health problem**
7	**Urinary tract infection**
8	**Fecal impaction**
9	**Symptoms of depression**
10	**Use of aids that inhibit freedom of movement**
11	**Participation in activities of interest**
12	**Participation in social activities**
13	**Decline in ADL**
14	**Weight loss**
15	**Falls**
16	**Hearing loss without the use of hearing aids**
17	*Established toilet routines*
18	*Bladder and bowel incontinence*
19	*Bedfast residents*
20	*Indwelling urinary catheter*

**Bold font**: consensus/accepted

*Italics:* NO consensus/rejected

*Abbreviation*: *ADL* Activities of Daily Living

^a^Ranking order according to the following procedure: 1) Consensus; 2) Median value; 3) Percent of rating between two adjoining values (6 and 7); 4) Interquartile range; 5) Range

When stratified by expert group, the ranking of the most important nursing-sensitive QI differed by group (Table 8). The scientific experts rated pain, dehydration, urinary tract infection, and use of aids that inhibit freedom of movement as the most important nursing-sensitive QIs. The clinical experts rated pain, pressure ulcers, and systematic medication review as the most important QIs, while the experts of experience rated systematic medication review, urinary tract infection, fecal impaction, and participation in activities of interest as the most important QIs.

**Table 8:** Consensus list of nursing-sensitive quality indicators in every expert group^a^

Scientific experts^b^		Clinical experts^c^		Experts of experience^d^	
**Ranking order**^**a**^** of the quality indicators**		**Ranking order**^**a**^** of the quality indicators**		**Ranking order**^**a**^** of the quality indicators**	
1	**Pain**	1	**Pain**	1	**Systematic medication review**
1	**Dehydration**	1	**Pressure ulcers**	1	**Urinary tract infection**
1	**Urinary tract infection**	1	**Systematic medication review**	1	**Fecal impaction**
1	**Use of aids that inhibit freedom of movement**	4	**Behavioral symptoms affecting others**	1	**Participation in activities of interest**
5	**Systematic medication review**	4	**Oral and/or dental health problem**	5	**Symptoms of depression**
6	**Pressure ulcers**	4	**Fecal impaction**	6	**Pressure ulcers**
6	**Behavioral symptoms affecting others**	7	**Symptoms of depression**	7	**Dehydration**
8	**Participation in social activities**	8	**Dehydration**	8	**Behavioral symptoms affecting others**
9	**Oral and/or dental health problem**	9	**Falls**	9	**Oral and/or dental health problem**
10	**Weight loss**	10	**Use of aids that inhibit freedom of movement**	10	**Hearing loss without the use of hearing aids**
11	**Decline in ADL**	11	**Participation in activities of interest**	11	**Pain**
12	**Participation in activities of interest**	12	**Decline in ADL**	12	**Participation in social activities**
13	**Falls**	12	**Weight loss**	13	**Decline in ADL**
13	**Hearing loss without the use of hearing aids**	14	**Participation in social activities**	14	**Weight loss**
15	**Symptoms of depression**	15	**Established toilet routines**	15	**Falls**
16	*Fecal impaction*	16	**Hearing loss without the use of hearing aids**	16	**Established toilet routines**
17	*Established toilet routines*	17	*Urinary tract infection*	17	**Use of aids that inhibit freedom of movement**
18	*Bladder and bowel incontinence*	18	*Bedfast residents*	18	*Bladder and bowel incontinence*
19	*Bedfast residents*	19	*Indwelling urinary catheter*	19	*Indwelling urinary catheter*
20	*Indwelling urinary catheter*	20	*Bladder and bowel incontinence*	20	*Bedfast residents*

**Bold font**: consensus

*Italics:* NO consensus

*Abbreviation*: *ADL* Activities of Daily Living

^a^Ranking order according to the following procedure: 1) Consensus; 2) Median value; 3) Percent of rating between two adjoining values (6 and 7); 4) Interquartile range; 5) Range

^b^Researchers

^c^Registered Nurses and Licensed Practical Nurses working in nursing homes

^d^Next-of-kin of nursing home residents

### Differences among participants and non-participants in the second Delphi round

We compared those who participated (*n* = 28) and those who did not participate (*n* = 16) in the second Delphi round and their rating of the 11 QIs for which consensus was not reached in the first Delphi round. Except for the QI ‘indwelling urinary catheter’, there was no significant difference in the rating of the QIs between participants and non-participants. The median value of the QI’indwelling urinary catheter’ was lower among those who participated in the second round (median value = 5) compared to those who did not participate in the second round (median value = 6; *p* = 0.002).

## Discussion

In this non-in-person, two-round, six-step modified Delphi study, we have described the process for developing a list of nursing-sensitive QIs for Norwegian NHs. A five-member project group drew up an initial list of 20 nursing-sensitive QIs from a preliminary list of 24 QIs from MDS/interRAI [46]. The project group rejected six of 24 QIs and added two new QIs. These QIs were: 1) ‘hearing loss without the use of hearing aids’, and 2) ‘systematic medication review’. In total, 44 and 28 experts rated nursing-sensitive QIs on a seven-point Likert scale in the first and second Delphi rounds, respectively. Consensus was based on median value and level of dispersion. The final list of nursing-sensitive QIs consisted of 16 QIs that were rated by all experts as important for measuring quality in NHs. The five QIs rated as most important were: 1) systematic medication review, 2) pressure ulcers, 3) behavioral symptoms affecting others, 4) pain, and 5) dehydration. There were four QIs for which consensus was not reached by all experts after the second round. These QIs were: 1) established toilet routines, 2) bladder and bowel incontinence, 3) bedfast residents, and 4) indwelling urinary catheter.

In Norwegian NHs, the physician has the main responsibility for medical treatment and care [63, 77]. As the aim of our study was to select and rate nursing-sensitive QIs, we did not include QIs from MDS/interRAI related to medical treatment of NH residents, such as ‘use of 9 or more different medications’ and ‘prevalence of antianxiety/hypnotic use’ [6, 46]. However, the project group considered ‘systematic medication review’ from the Norwegian quality assessment system IPLOS [14] as an important nursing-sensitive QI, as systematic medication reviews are usually initiated by nurses. Systematic medication review was ranked as the most important nursing-sensitive QI in the ‘all expert group’ and also among clinical experts and experts of experience. According to Norwegian guidelines, systematic medication reviews should be conducted at the time of NH admission and at least once per year, or when necessary for proper medical treatment. Medication reviews are often carried out in multidisciplinary teams with physicians, RNs, and pharmacists, and are based on observations from these professionals. Systematic medication reviews shall ensure the good quality of medical treatment of NH residents [14].

Randomized controlled trials in NHs have tested the effect of medication reviews and found that they lead to a reduced number of drugs [78, 79], falls [78], and costs [78], and improve quality of care for NH residents [80]. RNs working with direct patient care in NHs provide round-the-clock care [63]. Thus, they have the best opportunity to observe and assess therapeutic and adverse effects of medications, for example by monitoring and evaluating clinical status and vital signs [81]. These nursing observations must be communicated to the physician and the pharmacist [81]. In this way, RNs play an important part in pharmaceutical care, with a major impact on the quality of care [81]. A Norwegian study also concluded that RNs had an essential function in the multidisciplinary team that conducts medication reviews [77].

Few previous studies have selected and rated QIs from MDS [7] and interRAI for long-term care [9]. However, in line with our study, pressure ulcers, behavioral symptoms, and pain were ranked as one of the most important QIs in two Canadian studies using a modified Delphi technique [7] and a modified nominal group technique [9] to rate and prioritize QIs from MDS [7] and interRAI [9]. In the study by Sales et al. [9], the five top-ranked QIs were: 1) pressure ulcers, 2) worsening pain, 3) incontinence, 4) falls, and 5) little or no activity, while in the study by Estabrooks et al. [7], the top five ranked nursing-sensitive QIs were: 1) worsening pain, 1) antipsychotic use without psychosis, 3) pressure ulcers, 4) urinary tract infections, 5) physical restraint use; and 5) declining behavioral symptoms (shared first place and shared fifth place).

Comparing the results between the studies may be complicated due to the use of different methods to rank the QIs. The care context and participating experts included in the studies also differed [7, 9]. Sales et al. [9] included RNs, occupational therapists, dietitians, and one physician who voted for their top priorities from among 14 QIs. Each participant received three votes and the QIs were ranked according to the number of votes they received at the final meeting. Estabrooks et al. [7] included physicians, RNs, and policy makers, and the QIs were ranked according to their mean value. Neither of the studies included next-of-kin of NH residents or used the median value to achieve consensus for the QIs.

The QI ‘pain’ was rated as one of the most important nursing-sensitive QIs among both scientific experts and clinical experts. Previous studies have shown that a high proportion of NH residents with and without dementia have pain, and the prevalence varied between 32 and 80% in different studies [82–88]. The high prevalence of pain may be a consequence of a high proportion of NH residents having physical diagnoses and numerous potential sources of pain [2, 89]. The most common types of pain are musculoskeletal pain, neuropathic pain, pain related to coronary heart disease and cancer, orofacial pain, and surgical wound pain in post-acute care patients [89–93]. Pain in NH residents is linked to a decline in physical function [94, 95], mood disorders (depression and anxiety) [96], agitation [97], and poorer quality of life [82, 83, 98]. Thus, assessment of pain and severity of pain among NH residents at the time of admission and regularly thereafter is important in order to initiate non-pharmacological [99] and pharmacological treatment [92, 100], as necessary. However, ‘pain’ is not included as a QI in the Norwegian assessment system IPLOS [14], and a routine assessment of pain and pain severity should be implemented in the Norwegian NH-setting [82]. Pain assessment in MDS/interRAI is based on self-reporting, or is proxy-reported by nursing staff [46, 85]. Even though self-reporting is considered to be the ‘gold-standard’ in pain assessment [101], the MDS 2.0 pain assessment tool has been associated with underestimation of both pain and pain intensity among NH residents [85, 102–104], and especially among those with severe cognitive impairment [85, 103]. Thus, the validity of MDS regarding pain assessment is questioned [85, 103].

Assessment of pain in people with dementia with a self-reporting assessment tool may be challenging due to cognitive deficits, communication difficulties, and reduced self-reporting capacity [82, 85, 90]. Self-reporting increases the risk of underdiagnosis and undertreatment of pain in residents with dementia, and may be a trigger for neuropsychiatric symptoms such as agitation, aggression, psychosis, depression, apathy, and irritability [97, 105]. Thus, in residents with dementia, an observational behavioral pain scale such as MOBID-2 (Mobilization-Observation-Behavior-Intensity-Dementia Pain Scale) may be useful when assessing pain [90, 106]. Neuropsychiatric symptoms are prevalent in NH residents with dementia [97, 105, 107, 108], and several studies have shown an association between pain in residents with dementia and neuropsychiatric symptoms [97, 105, 109]. However, a causal pathway has yet to be determined [105]. Frequent signs of pain in residents with dementia are facial expressions (i.e., grimacing and frowning), verbalization (i.e., calling out and moaning), and defense postures (i.e., pushing and tensing) [82, 101, 105]. Some of these signs of pain may be mistaken as neuropsychiatric symptoms and treated with psychotropic drugs and restraints rather than thorough pain assessment and adequate pain treatment [82, 105, 107]. A number of studies [97, 105, 110] have shown that non-pharmacological and pharmacological treatment of pain in residents with dementia reduced both pain and neuropsychiatric symptoms. In our study, behavioral symptoms were also rated as one of the most important nursing-sensitive QIs in the ‘all expert group’. A reliable pain assessment followed by adequate pain treatment may also be a way to decrease behavioral symptoms among NH residents with dementia [97, 105, 110].

In our study, consensus was not reached concerning incontinence – neither in the ‘all expert group’ nor among scientific experts, clinical experts, or experts of experience. This finding was somewhat unexpected as the prevalence of both fecal and urinary incontinence is high among NH residents, and nursing interventions may prevent or ameliorate incontinence [44, 111–113]. In the already mentioned study by Sales et al. [9], incontinence was ranked as one of the most important QIs. However, similar to the finding in our study, incontinence was not included in the list of 13 nursing-sensitive QIs in the study by Estabrooks et al. [7]. Untreated urinary and fecal incontinence has been found to have serious adverse outcomes for NH residents, such as urinary tract infections, dermatitis, and higher mortality [114, 115]. Incontinence is also associated with reduced quality of life, low self-esteem, stigmatization, and feelings of social isolation among NH residents [112]. Therefore, there is no explanation for why consensus was not reached for this nursing-sensitive QI. However, the participants in our study may anticipate that incontinence among NH residents is an expected part of normal aging [116], where nursing interventions will have no effect on the prevalence. Further, incontinence is more prevalent in people with dementia than without [113, 117], and it is expected that people with dementia will develop incontinence as the disease progresses [118]. As a high proportion of NH residents have dementia (85%) and the severity of dementia among NH residents has increased in recent decades [1], nursing interventions may delay the onset of incontinence but not resolve it among residents with dementia.

Furthermore, consensus was not reached for ‘bedfast residents’ in any of the expert groups. In NHs, residents may be bedridden due to physical impairments, and acute and terminal illness [119, 120]. Being bedridden in the terminal phase is not considered to be a marker of low nursing quality, and these patients are normally excluded from the statistical analyses when measuring quality of care [121]. This information was not given to the participants in our study and may have affected the result that consensus was not achieved for this QI.

## Strengths and limitations

This study has several strengths. By using a Delphi method with two rounds, we were able to guide an opinion regarding nursing-sensitive QIs toward a final consensus [122]. Each participant could rate and express their views on each QI anonymously [123]. We also used controlled feedback of the results after the first round. Thus, the participants benefitted from seeing their own rating and the ratings of the other participants [42, 124]. According to Powell [42], the participants’ opportunity to revise previous ratings in light of the feedback from the first Delphi round is an important element in the process toward consensus. In addition, the anonymity allowed each participant to respond to the questionnaire without being biased by knowing the identities of other participants or being influenced by dominant individuals to reach consensus [59, 124].

A further strength of our study is that we considered the perspective of researchers, healthcare professionals, and next-of-kin of residents in NHs [42]. It is assumed that participants with different perspectives on a theme produce a higher quality of results than homogeneous groups [42]. However, we did not assess the residents’ perspectives, mainly because of the difficulty that residents with late-stage dementia would face participating in a non-in-person study. The view of the residents in NHs would also be of value [125–127], and should be explored further in another study, adjusting the methodology to the residents’ conditions.

Another strength of this study is that we used a method recommended by Røsvik et al. [71] and Hsu et al. [59] to define consensus, which was based on median value and level of dispersion [59, 71]. The use of a median score based on a Likert scale is strongly recommended as a measure of consensus in Delphi studies [59].

Despite the strengths of this Delphi study, there are limitations that should be considered.

The preliminary list of QIs was based on a narrative literature review and not a systematic review. This can be considered as a limitation of the present Delphi study. However, we do not assume that the preliminary list of QIs would have been very different even if we had conducted a systematic review in the first phase of the Delphi process, as the whole body of research was screened thoroughly, and all relevant QIs were included in the first phase. Further, the project group consisted only of RNs and did not include the next-of-kin of NH residents, and this could have biased the results. The inclusion and exclusion of QIs might have been different if next-of-kin had been part of the project group. However, the aim of the study was to explore QIs for clinical nursing, and it seemed adequate to only include RNs in the project group to ensure that nursing-sensitive areas were covered. Another limitation of the present Delphi study was that we a priori chose to use two Delphi rounds, and a third Delphi round might have led to different consensus results – for example, by increasing the accuracy of the different expert group’s decision making [42]. However, as each round was time-consuming, and a third round could possibly lead to participant fatigue and a further decline in participation rate [42], we chose to use two rounds. Two rounds have also been commonly used in previous Delphi studies evaluating QIs [6, 51, 52, 55, 70].

The Delphi method has been criticized by not allowing participants to meet in person and discuss the questions raised in the different rounds [49]. Another method that could have been used is the nominal group technique, which is a highly structured face-to-face meeting where information is gathered from relevant experts about a given theme/issue [128]. In a face-to-face meeting, the participants in our study could have been presented with theory and research in the field of nursing-sensitive QIs in NHs, and the participants could have shared opinions with each other. On the other hand, as the COVID-19 pandemic was ongoing when recruiting participants to this study, meetings in person could not be used, although meetings in person would have limited the broad geographic representation achieved by the use of an electronic questionnaire, especially among the researchers included in our study [124].

A limitation of the Delphi method is the lack of agreement on the size of the expert groups [56]. It is suggested that 10–15 participants would be sufficient in a homogeneous group [59]. Thus, our goal was to include 15 participants in every expert group. Although we did not reach that goal, we assume that the inclusion of 13 and 8 researchers, 17 and 10 healthcare professionals, and 14 and 10 next-of-kin in each Delphi round, respectively, was satisfactory.

The recruiting process for participants from NHs (healthcare professionals and next-of-kin) was prolonged. Thus, the time-period between the first and second Delphi round was longer than initially anticipated for the participants, and this may have contributed to the drop-out of 16 participants in the second round. Unfortunately, we do not know the exact reason for not participating in the second round. High drop-out in the second round may have affected the validity of the results. However, when comparing the rating among participants and non-participants in the second Delphi round, we found only one significant difference – namely, in the rating of the QI ‘indwelling urinary catheter’. Participants in the second Delphi round rated this QI lower (median value 5) than non-participants (median value 6). Although we might have a selection bias regarding this QI, we anticipate that the lower response rate in the second round did not influence the analysis results.

The participants in our study were selected for a purpose and not randomly selected, so representativeness is not assured [49]. Using a random sample might have strengthened the generalizability of the findings from the study [124]. If the same questionnaire was given to other participants, we may have received other consensus results on nursing-sensitive QIs [49]. However, in a Delphi study, it is not a criterion that the participants in the expert groups should be representative samples for statistical purposes [42]. Thus, the participants in our study were chosen on the basis of their qualifications [42] and their willingness to answer two rounds of questionnaires. Even so, a limitation was that only female researchers were included in the scientific expert group, and this might have introduced a selection bias. Male researchers may have evaluated the importance of the QIs differently to the female researchers.

In our study, nursing-sensitive QIs defined as process and outcome indicators were selected and rated [12]. Process and outcome indicators assess the actual nursing care and the outcome of the nursing care [12, 52]. However, to fully evaluate the quality of care in NHs, structure indicators also need to be evaluated [12], such as total staffing levels, ratio of RNs, ratio of unlicensed staff (care staff without healthcare education), registration of deviations, and physical characteristics of the NHs [12, 55, 75, 129–131]. However, structure indicators are not included in MDS/interRAI [11, 46]. Structure indicators might be considered as less nursing-sensitive, as healthcare professionals at the clinical level might have less influence on the organization of healthcare services and policy in the municipality [15]. Other important QIs that were not adequately captured in MDS/interRAI and not evaluated in this study, were quality of life, end-of-life care, dignity, autonomy, and patient participation [48, 56, 132, 133]. These QIs are particularly important in the vulnerable NH-population and should be included in an overall evaluation of quality of care. In addition, the participants were not allowed to suggest QIs during the Delphi rounds, and this is a limitation. QIs considered as very important by the researchers, the healthcare professionals, and the next-of-kin were not evaluated in our study.

## Implications

One way to increase the quality of care in NHs is to include nursing-sensitive QIs [13]*.* A nursing-sensitive QI can highlight areas in an NH that may be performed poorly and where the quality of care can be improved [9, 13]. Awareness of these areas may lead to better care processes and outcomes among the NH residents [9–11]. Thus, nursing-sensitive QIs are essential for clinical practice as they may have the greatest potential for functional improvement and slowing the functional decline among NH residents [7]. Nursing-sensitive QIs might also highlight areas where the quality of care is good or has been improving [134]. In this way, the QIs may uncover the care processes that have a positive effect on NH residents.

The consensus statements regarding nursing-sensitive QIs from this study can guide the clinical practice in Norwegian NHs [128]. The nursing-sensitive QIs that were rated as most important in this study should be prioritized for inclusion in quality assurance systems in Norwegian NHs and for increasing the quality of care. The challenge might be to implement QIs and use the QIs for continuous quality improvement and for evaluating nursing practice.

## Conclusions

The aim of this study was to use a modified Delphi method to rate and develop a final consensus list of nursing-sensitive QIs for Norwegian NHs. Scientific experts (researchers), clinical experts (healthcare professionals in NHs), and experts of experience (next-of-kin of NH residents) rated an initial list of 20 nursing-sensitive QIs. The final list of nursing-sensitive QIs consisted of 16 QIs, and the five most important QIs were: 1) systematic medication review, 2) pressure ulcers, 3) behavioral symptoms affecting others, 4) pain, and 5) dehydration. The final list of nursing-sensitive QIs could be included in Norwegian NHs’ quality systems, and guide nursing clinical practice and continuous quality improvement efforts.

### Supplementary Information

**Additional file 1.**

**Additional file 2.**

**Additional file 3.**

## Data Availability

The datasets generated and analyzed during the current study are available in anonymized form for researchers in cooperation with the data owners due to ethical restrictions by Norwegian law. If there is any need, please contact the corresponding author Kjerstin Tevik (kjerstin.e.tevik@ntnu.no).

## Abbreviations

ACOVE: Assessing Care of Vulnerable Elders
ADL: Activities of Daily Living
CQ: Consumer Quality index questionnaire
IBM SPSS: The International Business Machines Corporation Statistical Package for the Social Sciences
ICN: International Council of Nurses
interRAI LTCF: International Resident Assessment Instrument for Long-Term Care Facilities
IPLOS: In Norwegian: ‘Individbasert Pleie og Omorgsstatistikk’; In English: ‘Statistics linked to individual needs of care’
IQUARE: Impact of a Quality improvement questionnaire
ISM, NTNU: Department of Public Health and Nursing, Norwegian University of Science and Technology
LPN: Licensed Practical Nurse
MDS: Minimum Data Set
MOBID: Mobilization-Observation-Behavior-Intensity-Dementia Pain Scale
MSc: Master of Science
N: Number
NH: Nursing Home
PC: Personal Computer
PhD: Philosophiae doctor
PRISMA: Preferred Reporting Items for Systematic Reviews and Meta-Analyses
QI: Quality Indicator
RAI-MDS: Resident Assessment Instrument Minimum Data Set
RN: Registered Nurse
SERVQUAL: Service measurement tool for healthcare
