# Supplementary material for: Nursing-sensitive quality indicators for quality improvement in Norwegian nursing homes – a modified Delphi study
Source: BMC Health Serv Res. 2023 Oct 6;23:1068. doi: 10.1186/s12913-023-10088-4 (PMC10557356; doi:10.1186/s12913-023-10088-4)
Supplement: Supplementary file 1 — Additional file 1. [file 12913_2023_10088_MOESM1_ESM.docx]

**Additional file 1: Search strategies**

Search strategies (20.10.2020) in different databases for the article “Nursing sensitive quality indicators for quality improvement in Norwegian nursing homes – A modified Delphi study”.

| **Key words / MeSH terms** | **Database** | **Combinations** | **Results** |
| --- | --- | --- | --- |
| **1) «quality indicator*» MeSH: indicators, health care+key word** | OVID Medline |  | 23779  2020: 953  2010-2020: 15435  2000-2009: 7024  1990-1999: 1261  1980-1989: 0 |
| **2) Municipality OR**  **Home Care Services/Home Nursing (MeSH) OR**  **“Nursing home” OR**  **“Care home” OR**  **“Disabled persons”/Intellectual Disability (MeSH) OR**  **Mental health Services (MeSH) OR**  **Manage* OR**  **Professional Autonomy (MeSH) OR**  **Work environment** | OVID Medline |  | 1855325 |
| **Search 1 AND search 2** | OVID Medline | Patient population + intervention | 7051 |

| **Key words /MeSH terms** | **Database** | **Combinations** | **Results** |
| --- | --- | --- | --- |
| **3) “quality indicator”** | OVID comb. Search: PsycInfo+ Embase+Medline+Ovid Books | Limited to  Elderly and humans only.  Duplicates removed. | (37842)  8016  2020: (229)173  2010-2020: (5610)4136  2000-2009: (2182)1579  1990-1999: (217)168  1985-1989: (2)1 |
| **4) Municipality OR**  **Home Care Services/Home Nursing OR**  **“Nursing home” OR**  **“Care home” OR**  **“Disabled persons”/Intellectual Disability OR**  **Mental health Services OR**  **Manage* OR**  **Professional Autonomy OR**  **Work environment** | OVID comb. Search: PsycInfo+ Embase+Medline+Ovid Books |  | 5005225 |
| **Search 3 (without limits) AND search 4** | OVID comb. Search: PsycInfo+ Embase+Medline+Ovid Books |  | 12723 |
| **Search 3 (with limits) AND search 4*** | OVID comb. Search: PsycInfo+ Embase+Medline+Ovid Books | Duplicates removed. | (2843) 2180  2010-2020: 1406  2000-2009: 700  1990-1999: 73  1985-1989: 0 |

| **Key words / MeSH terms** | **Database** | **Combinations** | **Results** |
| --- | --- | --- | --- |
| **Governance** | OVID comb. Search: PsycInfo+ Embase+Medline |  | 7045 |
| **Accountability** | OVID comb. Search: PsycInfo+ Embase+Medline |  | 7850 |
| **“New Public Management”** | OVID comb. Search: PsycInfo+ Embase+Medline | Duplicates removed | (689)507 |
| **Benchmark*** | OVID comb. Search: PsycInfo+ Embase+Medline | Duplicates removed | (4559)3899 |
| **Audit** | OVID comb. Seach: PsycInfo+ Embase+Medline |  | 22636 |
| **“Performance indicator*”** | OVID comb. Search: PsycInfo+ Embase+Medline |  | 11206 |
| **Accreditation** | OVID comb. Search: PsycInfo+ Embase+Medline |  | 9843 |
| **Certification** | OVID comb. Search: PsycInfo+ Embase+Medline |  | 11998 |
| **Municipality OR**  **Home Care Services/Home Nursing OR**  **“Nursing home” OR**  **“Care home” OR**  **“Disabled persons”/Intellectual Disability OR**  **Mental health Services OR**  **Manage* OR**  **Professional Autonomy OR**  **Work environment** | OVIDMedline | AND «New Public Management» from the combination search. | 151  *Transferred to EndNote |
| **Municipality OR**  **Home Care Services/Home Nursing OR**  **“Nursing home” OR**  **“Care home” OR**  **“Disabled persons”/Intellectual Disability OR**  **Mental health Services OR**  **Manage* OR**  **Professional Autonomy OR**  **Work environment** | OVIDMedline | AND “Performance indicator*” from the combination search. | 1287  *Transferred to EndNote |

| **Keywords / MeSH terms** | **Database** | **Combinations** | **Results** |
| --- | --- | --- | --- |
| **(“quality indicator*” “Health care”)** | Web of Science |  | 2313 |
| **(“quality indicator*” “Health care”)** | Web of Science | Municipality OR  Home Care Services OR  “Nursing home” OR  “Care home” OR  “Disabled persons”/Intellectual Disability OR  Mental health Services OR  Manage* OR  Professional Autonomy OR  Work environment | 8  3  59  4  1  4  35  823  1  7 |
