# Supplementary material for: Nursing-sensitive quality indicators for quality improvement in Norwegian nursing homes – a modified Delphi study
Source: BMC Health Serv Res. 2023 Oct 6;23:1068. doi: 10.1186/s12913-023-10088-4 (PMC10557356; doi:10.1186/s12913-023-10088-4)
Supplement: Supplementary file 2 — Additional file 2. [file 12913_2023_10088_MOESM2_ESM.docx]

Records identified through database searching (n = 5530)

## Identification

Records identified through searching in Rayyan with predefined terms
(NH, long term care, validity, MDS) (n = 754)

Additional records identified through other sources
(n = 49)

Records screened
(n = 803)

Records excluded
(n = 528)

## Screening

Full-text articles assessed for eligibility
(n = 275)

Full-text articles excluded, with reasons (n = 203)

- Not used MDS/interRAI

LTCF (n = 69)

- Focus on economy, organi-

zational, and environmental

factors (n = 70)

- Methodologicial/theoretical

articles (n = 27)

- Not conducted in nursing

homes (n = 20)

- Not conducted in a Western

country (n = 11)

- Not focus on nursing

sensitive QIs (n = 6)

- Other reasons (n = 6)

## Eligibility

Studies included in

Additional file 3
(n = 72)

## Included

**Additional file 2:** Flow diagram depicting study identified, screened, and included in Additional file 3 (1)

Abbreviations: interRAI LTCF = international Resident Assessment Instrument for Long Term Care Facilities; MDS = Minimum Data Set, QI = Quality Indicator; NH = nursing home

1. Moher D, Liberati A, Tetzlaff J, Altman DG. Preferred reporting items for systematic reviews and meta-analyses: the PRISMA statement. PLoS Med. 2009;6(7):e1000097. <https://doi.org/10.1371/journal.pmed.1000097>
