# Supplementary material for: Nursing-sensitive quality indicators for quality improvement in Norwegian nursing homes – a modified Delphi study
Source: BMC Health Serv Res. 2023 Oct 6;23:1068. doi: 10.1186/s12913-023-10088-4 (PMC10557356; doi:10.1186/s12913-023-10088-4)
Supplement: Supplementary file 3 — Additional file 3. [file 12913_2023_10088_MOESM3_ESM.docx]

**Additional file 3: A preliminary list of quality indicators based on a literature review**

| **Domain** | **Quality indicators** | **Process/Outcome** | **References** |
| --- | --- | --- | --- |
| **1. Cognitive functioning** | 1.Incidence of cognitive impairment | Outcome | (1-13) |
| **2. Behavioural and emotional patterns** | 2. Prevalence of behavioral symptoms affecting others | Outcome | (1, 2, 5, 6, 8, 10, 13-15) |
|  | 3. Prevalence of symptoms of depression | Outcome | (1-3, 5, 6, 8, 10, 11, 13-24) |
|  | 4. Prevalence of symptoms of depression without antidepressant therapy | Process/  Outcome | (2, 5, 6, 8, 10, 11, 13-15, 17, 24-27) |
| **3. Physical functioning** | 5. Prevalence of bedfast residents | Outcome | (1, 2, 5, 6, 8, 10, 11, 13-15, 17, 28-30) |
|  | 6. Incidence of decline in ADLs (activities of daily living) | Outcome | (1-3, 5, 6, 10-13, 25, 26, 28, 31-39) |
| **4. Elimination and continence** | 7. Prevalence of bladder/bowel incontinence | Outcome | (2, 3, 5, 6, 10-15, 25-27, 30, 35, 39-44) |
|  | 8. Prevalence of indwelling catheters | Process | (2, 3, 5, 6, 8, 10, 11, 13-15, 17, 22, 23, 25, 28, 31, 32, 35, 45, 46) |
|  | 9. Prevalence of fecal impaction | Outcome | (2, 5, 6, 8, 10, 11, 14, 15, 17) |
|  | 10. Prevalence of toilet assistance program | Process | (47) |
| **5. Infection control** | 11. Prevalence of urinary tract infections | Outcome | (6, 11, 13, 15, 22, 23, 30, 35, 48) |
| **6. Accidents/ Health condition** | 12. Prevalence of falls | Outcome | (2, 3, 5, 6, 8, 10, 11, 13-15, 17, 27, 30-32, 34, 49) |
|  | 13. Prevalence of pain | Outcome | (3, 8, 21, 27, 28, 31-36, 50-59) |
| **7. Nutrition and eating** | 14. Prevalence of weight loss | Outcome | (1-3, 5, 6, 8, 10, 14, 15, 17, 26, 28, 31-33, 60, 61) |
|  | 15. Prevalence of tube feeding | Prosess | (2, 3, 5, 8, 10, 11, 13-15, 17, 26, 31, 32, 45, 46) |
|  | 16. Prevalence of dehydration | Outcome | (2, 5, 8, 10, 11, 13-15, 17, 27, 30) |
|  | 17. Prevalence of oral and dental health problem | Prosess | (1, 8, 27, 62-64) |
| **8. Skin care** | 18. Prevalence of pressure ulcers | Outcome | (1-3, 5, 6, 8, 14, 15, 17, 25, 26, 28, 30-33, 35, 36, 45, 46, 57, 60, 65-68) |
| **9. Clinical management** | 19. Use of 9 or more different medications | Process | (2, 5, 6, 10, 11, 13, 14, 15, 17, 27, 30) |
| **10. Psychotropic drug use** | 20. Prevalence of antipsychotic use in the  absence of psychotic and related conditions | Process | (2, 5, 6, 10, 13-15, 17, 22, 23, 25, 32, 69, 70) |
|  | 21. Prevalence of antianxiety/hypnotic use | Process | (2, 5, 6, 10, 11, 13-15, 17, 27, 71) |
| **11. Quality of life** | 22. Prevalence of little or no activity | Process | (2, 3, 5, 6, 8, 10, 11, 13, 14, 15, 17, 27, 30, 46) |
|  | 23. Prevalence of little or no social activity | Process | (31) |
|  | 24. Prevalence of physical restraints | Process | (2, 3, 5, 6, 8, 10, 11, 14, 15, 17, 26, 28, 31-33, 36, 45, 57, 60, 65, 72) |

*Main references: Zimmerman 2003 et al.: Improving nursing home quality of care through outcomes data: the MDS quality indicators (13); Morris et al: interRAI Long-Term Care Facilities (LTCF). Assessment Form and User's Manual. Version 9.1. Norwegian version. Washington, DC: interRAI, 2012 (73).

**References**

1. **Blaum** CS, O'Neill EF, Clements KM, Fries BE, Fiatarone MA. Validity of the minimum data set for assessing nutritional status in nursing home residents. Am J Clin Nutr. 1997;66(4):787-94. <https://doi.org/10.1093/ajcn/66.4.787>

2. **Castl**e NG, Ferguson JC. What is nursing home quality and how is it measured? Gerontologist.

2010;50(4):426-42. <https://doi.org/10.1093/geront/gnq052>

3. **Frijters** DH, van der Roest HG, Carpenter IG, Finne-Soveri H, Henrard JC, Chetrit A, et al. The calculation of quality indicators for long term care facilities in 8 countries (SHELTER project). BMC Health Serv Res. 2013;13:138. <https://doi.org/10.1186/1472-6963-13-138>

4. **Gruber-Baldini** AL, Zimmerman SI, Mortimore E, Magaziner J. The validity of the minimum data set in measuring the cognitive impairment of persons admitted to nursing homes. J Am Geriatr Soc. 2000;48(12):1601-6. <https://doi.org/10.1111/j.1532-5415.2000.tb03870.x>

5. **Hutchinson** AM, Milke DL, Maisey S, Johnson C, Squires JE, Teare G, et al. The Resident Assessment Instrument-Minimum Data Set 2.0 quality indicators: a systematic review. BMC Health Serv Res. 2010;10:166. <https://doi.org/10.1186/1472-6963-10-166>

6. **Laine** J, Finne-Soveri UH, Björkgren M, Linna M, Noro A, Häkkinen U. The association between quality of care and technical efficiency in long-term care. Int J Qual Health Care. 2005;17(3):259-67. <https://doi.org/10.1093/intqhc/mzi032>

7. **Morris** JN, Fries BE, Mehr DR, Hawes C, Phillips C, Mor V, et al. MDS Cognitive Performance Scale. J Gerontol. 1994;49(4):M174-82. <https://doi.org/10.1093/geronj/49.4.m174>

8. **Nakrem** S, Vinsnes AG, Harkless GE, Paulsen B, Seim A. Nursing sensitive quality indicators for nursing home care: international review of literature, policy and practice. Int J Nurs Stud. 2009;46(6):848-57. <https://doi.org/10.1016/j.ijnurstu.2008.11.005>

9. **Paquay** L, De Lepeleire J, Schoenmakers B, Ylieff M, Fontaine O, Buntinx F. Comparison of the diagnostic accuracy of the Cognitive Performance Scale (Minimum Data Set) and the Mini-Mental State Exam for the detection of cognitive impairment in nursing home residents. Int J Geriatr Psychiatry. 2007;22(4):286-93. <https://doi.org/10.1136/bmjopen-2013-004488>

10. **Rantz** MJ, Petroski GF, Madsen RW, Mehr DR, Popejoy L, Hicks LL, et al. Setting thresholds for quality indicators derived from MDS data for nursing home quality improvement reports: an update. Jt Comm J Qual Improv. 2000;26(2):101-10. <https://doi.org/10.1016/s1070-3241(00)26008-2>

11. **Rantz** MJ, Popejoy L, Petroski GF, Madsen RW, Mehr DR, Zwygart-Stauffacher M, et al. Randomized clinical trial of a quality improvement intervention in nursing homes. Gerontologist. 2001;41(4):525-38. <https://doi.org/10.1093/geront/41.4.525>

12. **Wu** N, Mor V, Roy J. Resident, nursing home, and state factors affecting the reliability of Minimum Data Set quality measures. Am J Med Qual. 2009;24(3):229-40. <https://doi.org/10.1097/01.mlr.0000173595.66356.12>

13. **Zimmerman** DR. Improving nursing home quality of care through outcomes data: the MDS quality indicators. Int J Geriatr Psychiatry. 2003;18(3):250-7. <https://doi.org/10.1002/gps.820>

14. **Hjaltadóttir** I, Hallberg IR, Ekwall AK. Thresholds for minimum data set quality indicators developed and applied in icelandic nursing homes. J Nurs Care Qual. 2012;27(3):266-76. <https://doi.org/10.1097/NCQ.0b013e3182493646>

15. **Jensdóttir** AB, Rantz M, Hjaltadóttir I, Gudmundsdòttir H, Rook M, Grando V. International comparison of quality indicators in United States, Icelandic and Canadian nursing facilities. Int Nurs Rev. 2003;50(2):79-84. <https://doi.org/10.1046/j.1466-7657.2003.00163.x>

16. **Heiser** D. Depression Identification in the Long-Term Care Setting. Clinical Gerontologist. 2004;27(4):3-18. <https://doi.org/10.1300/J018v27n04_02>

17. **Hjaltadóttir** I, Ekwall AK, Nyberg P, Hallberg IR. Quality of care in Icelandic nursing homes measured with Minimum Data Set quality indicators: retrospective analysis of nursing home data over 7 years. Int J Nurs Stud. 2012;49(11):1342-53. <https://doi.org/10.1016/j.ijnurstu.2012.06.004>

18. **Huang** Y, Carpenter I. Identifying elderly depression using the Depression Rating Scale as part of comprehensive standardised care assessment in nursing homes. Aging Ment Health. 2011;15(8):1045-51. <https://doi.org/10.1080/13607863.2011.583626>

19. **Schnelle** JF, Wood S, Schnelle ER, Simmons SF. Measurement sensitivity and the Minimum Data Set depression quality indicator. Gerontologist. 2001;41(3):401-5. <https://doi.org/10.1093/geront/41.3.401>

20. **Simmons** SF, Cadogan MP, Cabrera GR, Al-Samarrai NR, Jorge JS, Levy-Storms L, et al. The minimum data set depression quality indicator: does it reflect differences in care processes? Gerontologist. 2004;44(4):554-64. <https://doi.org/10.1093/geront/44.4.554>

21. **Temkin-Greener** H, Ladwig S, Ye Z, Norton SA, Mukamel DB. Improving palliative care through teamwork (IMPACTT) in nursing homes: Study design and baseline findings. Contemp Clin Trials. 2017;56:1-8. <https://doi.org/10.1016/j.cct.2017.01.011>

22. **Xu** D, Kane RL, Shippee T, Lewis TM. Identifying Consistent and Coherent Dimensions of Nursing Home Quality: Exploratory Factor Analysis of Quality Indicators. J Am Geriatr Soc. 2016;64(12):e259-e64. <https://doi.org/10.1111/jgs.14562>

23. **Xu** D, Kane R, Arling G. Relationship between nursing home quality indicators and potentially preventable hospitalisation. BMJ Qual Saf. 2019;28(7):524-33. <https://doi.org/10.1136/bmjqs-2018-008924>

24. **Zisselman** MH, Warren RS, Cutillo-Schmitter T, Denman SJ. Challenging the quality of the quality indicator, "depression without treatment". J Am Med Dir Assoc. 2002;3(2):41-5.

25. **Arling** G, Karon SL, Sainfort F, Zimmerman DR, Ross R. Risk adjustment of nursing home quality indicators. Gerontologist. 1997;37(6):757-66. <https://doi.org/10.1093/geront/37.6.757>

26. **Berg** K, Mor V, Morris J, Murphy KM, Moore T, Harris Y. Identification and evaluation of existing nursing homes quality indicators. Health Care Financ Rev. 2002;23(4):19-36. Available from: [Identification and Evaluation of Existing Nursing Homes Quality Indicators.: EBSCOhost](https://web.s.ebscohost.com/ehost/pdfviewer/pdfviewer?vid=0&sid=b5e6aed1-fcf5-4193-9c39-2643815b4784%40redis) (18.01.2023)

27. **Sales** A, O'Rourke HM, Draper K, Teare GF, Maxwell C. Prioritizing information for quality improvement using resident assessment instrument data: experiences in one canadian province. Healthc Policy. 2011;6(3):55-69. <https://doi.org/10.12927/hcpol.2011.22221>

28. **Arling** G, Lewis T, Kane RL, Mueller C, Flood S. Improving quality assessment through multilevel

modeling: the case of nursing home compare. Health Serv Res. 2007;42(3 Pt 1):1177-99.

<https://doi.org/10.1111/j.1475-6773.2006.00647.x>

29. **Bates-Jensen** BM, Alessi CA, Cadogan M, Levy-Storms L, Jorge J, Yoshii J, et al. The Minimum Data Set bedfast quality indicator: differences among nursing homes. Nurs Res. 2004;53(4):260-72. <https://doi.org/10.1097/00006199-200407000-00009>

30. **Moty** C, Barberger-Gateau P, De Sarasqueta AM, Teare GF, Henrard JC. Risk adjustment of quality indicators in French long term care facilities for elderly people. A preliminary study. Rev Epidemiol Sante Publique. 2003;51(3):327-38.

31. **Boorsma** M, Frijters DH, Knol DL, Ribbe ME, Nijpels G, van Hout HP. Effects of multidisciplinary integrated care on quality of care in residential care facilities for elderly people: a cluster randomized trial. Cmaj. 2011;183(11):E724-32. <https://doi.org/10.1503/cmaj.101498>

32. **Estabrooks** CA, Knopp-Sihota JA, Norton PG. Practice sensitive quality indicators in RAI-MDS 2.0 nursing home data. BMC Res Notes. 2013;6:460. <https://doi.org/10.1186/1756-0500-6-460>

33. **Harris** Y, Clauser SB. Achieving improvement through nursing home quality measurement. Health Care Financ Rev. 2002;23(4):5-18. Available from: [11049.Harris (nih.gov)](https://www.ncbi.nlm.nih.gov/pmc/articles/PMC4194764/pdf/hcfr-23-4-005.pdf)

34. **Hirdes** JP, Ljunggren G, Morris JN, Frijters DH, Finne Soveri H, Gray L, et al. Reliability of the

interRAI suite of assessment instruments: a 12-country study of an integrated health information system. BMC

Health Serv Res. 2008;8:277. <https://doi.org/10.1186/1472-6963-8-277>

35. **Morris** JN, Moore T, Jones R, Mor V, Joseph A, Berg K, et al. Validation of long-term and post-acute care quality indicators. Centers for Medicare and Medicaid Services. Office of Clinical Standard of Quality; 2003. CMS Contract No: 500-95-0062/ T.O. #4. Available from: [Validation of Long-Term and Post-Acute Care Quality Indicators (cms.gov)](https://www.cms.gov/Medicare/Quality-Initiatives-Patient-Assessment-Instruments/NursingHomeQualityInits/Downloads/NHQIexecsummary_finaldraft.pdf) (18.01.2023)

36. **Mukame**l DB, Weimer DL, Spector WD, Ladd H, Zinn JS. Publication of quality report cards and

trends in reported quality measures in nursing homes. Health Serv Res. 2008;43(4):1244-62.

<https://doi.org/10.1111/j.1475-6773.2007.00829.x>

37. **Phillips** CD, Shen R, Chen M, Sherman M. Evaluating nursing home performance indicators: an illustration exploring the impact of facilities on ADL change. Gerontologist. 2007;47(5):683-9. <https://doi.org/10.1093/geront/47.5.683>

38. **Phillips** CD, Chen M, Sherman M. To what degree does provider performance affect a quality indicator? The case of nursing homes and ADL change. Gerontologist. 2008;48(3):330-7. <https://doi.org/10.1093/geront/47.5.683>

39. **Rantz** MJ, Zwygart-Stauffacher M, Hicks L, Mehr D, Flesner M, Petroski GF, et al. Randomized multilevel intervention to improve outcomes of residents in nursing homes in need of improvement. J Am Med Dir Assoc. 2012;13(1):60-8. <https://doi.org/10.1016/j.jamda.2011.06.012>

40. **Blekken** LE, Vinsnes AG, Gjeilo KH, Norton C, Mørkved S, Salvesen Ø, et al. Exploring faecal incontinence in nursing home patients: a cross-sectional study of prevalence and associations derived from the Residents Assessment Instrument for Long-Term Care Facilities. J Adv Nurs. 2016;72(7):1579-91. <https://doi.org/10.1111/jan.12932>

41. **Li** Y, Schnelle J, Spector WD, Glance LG, Mukamel DB. The "Nursing Home Compare" measure of urinary/fecal incontinence: cross-sectional variation, stability over time, and the impact of case mix. Health Serv Res. 2010;45(1):79-97. <https://doi.org/10.1111/j.1475-6773.2009.01061.x>

42. **Morgan** C, Endozoa N, Paradiso C, McNamara M, McGuire M. Enhanced toileting program decreases incontinence in long term care. Jt Comm J Qual Patient Saf. 2008;34(4):206-8. <https://doi.org/10.1016/s1553-7250(08)34026-4>

43. **Mukamel** DB, Watson NM, Meng H, Spector WD. Development of a risk-adjusted urinary incontinence outcome measure of quality for nursing homes. Med Care. 2003;41(4):467-78. <https://doi.org/10.1097/01.Mlr.0000053227.95476.02>

44. **Schnelle** JF, Cadogan MP, Yoshii J, Al-Samarrai NR, Osterweil D, Bates-Jensen BM, et al. The minimum data set urinary incontinence quality indicators: do they reflect differences in care processes related to incontinence? Med Care. 2003;41(8):909-22. <https://doi.org/10.1097/00005650-200308000-00005>

45. **McGarry** BE, Joyce NR, McGuire TG, Mitchell SL, Bartels SJ, Grabowski DC. Association between High Proportions of Seriously Mentally Ill Nursing Home Residents and the Quality of Resident Care. J Am Geriatr Soc. 2019;67(11):2346-52. <https://doi.org/10.1111/jgs.16080>

46. **Mor** V, Angelelli J, Jones R, Roy J, Moore T, Morris J. Inter-rater reliability of nursing home quality

indicators in the U.S. BMC Health Serv Res. 2003;3(1):20. <https://doi.org/10.1186/1472-6963-3-20>

47. **Schnelle** JF, Cadogan MP, Grbic D, Bates-Jensen BM, Osterweil D, Yoshii J, et al. A standardized quality assessment system to evaluate incontinence care in the nursing home. J Am Geriatr Soc. 2003;51(12):1754-61. <https://doi.org/10.1046/j.1532-5415.2003.51560.x>

48. **Stevenson** KB, Moore JW, Sleeper B. Validity of the minimum data set in identifying urinary tract infections in residents of long-term care facilities. J Am Geriatr Soc. 2004;52(5):707-11. <https://doi.org/10.1111/j.1532-5415.2004.52206.x>

49. **Hill-Westmoreland** EE, Gruber-Baldini AL. Falls documentation in nursing homes: agreement between the minimum data set and chart abstractions of medical and nursing documentation. J Am Geriatr Soc. 2005;53(2):268-73. <https://doi.org/10.1111/j.1532-5415.2005.53113.x>

50. **Burfield** A. Cohort Study Of Pain Behaviors In The Elderly Residing In Skilled Nursing Care [Doctoral Dissertation]: University of Central Florida; 2009. Available from: [Cohort Study Of Pain Behaviors In The Elderly Residing In Skilled Nursing Care (ucf.edu)](https://stars.library.ucf.edu/cgi/viewcontent.cgi?article=5018&context=etd) (18.01.2023)

51. **Cadogan** MP, Schnelle JF, Yamamoto-Mitani N, Cabrera G, Simmons SF. A minimum data set

prevalence of pain quality indicator: is it accurate and does it reflect differences in care processes? J Gerontol A

Biol Sci Med Sci. 2004;59(3):281-5. <https://doi.org/10.1093/gerona/59.3.m281>

52. **Cadogan** MP, Schnelle JF, Al-Sammarrai NR, Yamamoto-Mitani N, Cabrera G, Osterweil D, et al. A

standardized quality assessment system to evaluate pain detection and management in the nursing home. J Am

Med Dir Assoc. 2006;7(3 Suppl):S11-9, s0. <https://doi.org/10.1016/j.jamda.2005.12.011>

53. **Fries** BE, Simon SE, Morris JN, Flodstrom C, Bookstein FL. Pain in U.S. nursing homes: validating a pain scale for the minimum data set. Gerontologist. 2001;41(2):173-9. <https://doi.org/10.1093/geront/41.2.173>

54. **Guion** V, De Souto Barreto P, Sourdet S, Rolland Y. Effect of an Educational and Organizational

Intervention on Pain in Nursing Home Residents: A Nonrandomized Controlled Trial. J Am Med Dir Assoc.

2018;19(12):1118-23.e2. <https://doi.org/10.1016/j.jamda.2018.09.031>

55. **Hirdes** JP, Retalic T, Muskat C, Morris JN, Katz PR. The Seniors Quality Leap Initiative (SQLI): An International Collaborative to Improve Quality in Long-Term Care. J Am Med Dir Assoc. 2020;21(12):1931-6. <https://doi.org/10.1016/j.jamda.2020.07.024>

56. **Horner** JK, Hanson LC, Wood D, Silver AG, Reynolds KS. Using quality improvement to address pain management practices in nursing homes. J Pain Symptom Manage. 2005;30(3):271-7. <https://doi.org/10.1016/j.jpainsymman.2005.03.014>

57. **Mukamel** DB, Glance LG, Li Y, Weimer DL, Spector WD, Zinn JS, et al. Does risk adjustment of the CMS quality measures for nursing homes matter? Med Care. 2008;46(5):532-41. <https://doi.org/10.1097/MLR.0b013e31816099c5>

58. **Pimentel** CB, Briesacher BA, Gurwitz JH, Rosen AB, Pimentel MT, Lapane KL. Pain management in nursing home residents with cancer. J Am Geriatr Soc. 2015;63(4):633-41. <https://doi.org/10.1111/jgs.13345>

59. **Wu** N, Miller SC, Lapane K, Roy J, Mor V. Impact of cognitive function on assessments of nursing home residents' pain. Med Care. 2005;43(9):934-9. <https://doi.org/10.1097/01.mlr.0000173595.66356.12>

60. **Carter** MW, Porell FW. Nursing home performance on select publicly reported quality indicators and resident risk of hospitalization: grappling with policy implications. J Aging Soc Policy. 2006;18(1):17-39. <https://doi.org/10.1300/J031v18n01_02>

61. **Lorin**i C, Porchia BR, Pieralli F, Bonaccorsi G. Process, structural, and outcome quality indicators of nutritional care in nursing homes: a systematic review. BMC Health Serv Res. 2018;18(1):43. <https://doi.org/10.1186/s12913-018-2828-0>

62. **Hoben** M, Poss JW, Norton PG, Estabrooks CA. Oral/dental items in the resident assessment instrument - minimum Data Set 2.0 lack validity: results of a retrospective, longitudinal validation study. Popul Health Metr. 2016;14:36. <https://doi.org/10.1186/s12963-016-0108-y>

63. **Krausch-Hofmann** S, De Almeida Mello J, Declerck D, Declercq A, De Lepeleire J, Tran TD, et al. The oral health-related section of the interRAI: Evaluation of test content validity by expert rating and assessment of potential reasons for inaccurate assessments based on focus group discussions with caregivers. Gerodontology. 2019;36(4):382-94. <https://doi.org/10.1111/ger.12421>

64. **Thai** PH, Shuman SK, Davidson GB. Nurses' dental assessments and subsequent care in Minnesota

nursing homes. Spec Care Dentist. 1997;17(1):13-8. <https://doi.org/10.1111/j.1754-4505.1997.tb00530.x>

65. **Baier** R, Butterfield K, Patry G, Harris Y, Gravenstein S. Identifying star performers: the relationship

between ambitious targets and nursing home quality improvement. J Am Geriatr Soc. 2009;57(8):1498-503.

<https://doi.org/10.1111/j.1532-5415.2009.02362.x>

66. **Bates-Jensen** BM, Cadogan M, Jorge J, Schnelle JF. Standardized quality-assessment system to evaluate pressure ulcer care in the nursing home. J Am Geriatr Soc. 2003;51(9):1194-202. <https://doi.org/10.1046/j.1532-5415.2003.51402.x>

67. **Berlowitz** DR, Bezerra HQ, Brandeis GH, Kader B, Anderson JJ. Are we improving the quality of

nursing home care: the case of pressure ulcers. J Am Geriatr Soc. 2000;48(1):59-62.

<https://doi.org/10.1111/j.1532-5415.2000.tb03029.x>

68. **Berlowitz** DR, Christiansen CL, Brandeis GH, Ash AS, Kader B, Morris JN, et al. Profiling nursing homes using Bayesian hierarchical modeling. J Am Geriatr Soc. 2002;50(6):1126-30. <https://doi.org/10.1046/j.1532-5415.2002.50272.x>

69. **Hirdes** JP, Major J, Didic S, Quinn C, Mitchell L, Chen J, et al. A Canadian Cohort Study to Evaluate the Outcomes Associated with a Multicenter Initiative to Reduce Antipsychotic Use in Long-Term Care Homes. J Am Med Dir Assoc. 2020;21(6):817-22. <https://doi.org/10.1016/j.jamda.2020.04.004>

70. **Norton** PG, Murray M, Doupe MB, Cummings GG, Poss JW, Squires JE, et al. Facility versus unit level reporting of quality indicators in nursing homes when performance monitoring is the goal. BMJ Open. 2014;4(2):e004488. <https://doi.org/10.1136/bmjopen-2013-004488>

71. **Lix** LM, Yan L, Blackburn D, Hu N, Schneider-Lindner V, Shevchuk Y, et al. Agreement between administrative data and the Resident Assessment Instrument Minimum Dataset (RAI-MDS) for medication use in long-term care facilities: a population-based study. BMC Geriatr. 2015;15:24. <https://doi.org/10.1186/s12877-015-0023-2>

72. **Castle** NG. Mental health outcomes and physical restraint use in nursing homes {private}. Adm Policy

Ment Health. 2006;33(6):696-704. <https://doi.org/10.1007/s10488-006-0080-0>

73. **Morris** JN, Belleville-Taylor P, Fries BE, Hawes C, Murphy K, Mor V, et al. interRAI Long-Term Care Facilities (LTCF) Assessment Form and User's Manual. Version 9.1. Norwegian version. utgave. Washington, DC: interRAI, 2012
